# Supplementary material for: Unified picture of vibrational relaxation of OH stretch at the air/water interface
Source: Nat Commun. 2024 Feb 10;15:1258. doi: 10.1038/s41467-024-45388-8 (PMC10858864; doi:10.1038/s41467-024-45388-8)
Supplement: Supplementary file 1 — Supplementary Information [file 41467_2024_45388_MOESM1_ESM.pdf]

Supplementary Information for

Unified Picture of Vibrational Relaxation of OH Stretch at the Air/Water Interface

Woongmo Sung[1], Ken-ichi Inoue[1], Satoshi Nihonyanagi[1, 2], and Tahei Tahara[1, 2*]

[1] Molecular Spectroscopy Laboratory, RIKEN, 2-1 Hirosawa, Wako, Saitama 351-0198, Japan

[2] Ultrafast Spectroscopy Research Team, RIKEN Center for Advanced Photonics (RAP), 2-1 Hirosawa, Wako, Saitama 351-0198, Japan

Supplementary Notes

1. Instrumental response of the TR-HD-VSFG setup
2. Time-resolved $\Delta Im\chi^{(2)}$ spectra at long delay times after IR excitation
3. Details of fitting analysis of the temporal traces of the $\Delta Im\chi^{(2)}$ signals
4. Singular value decomposition (SVD) analysis of the time-resolved $\Delta Im\chi^{(2)}$ spectra in the excited-state HB OH stretch band region
5. TR-HD-VSFG experiments using broadband excitation in the ground-state bleach region and singular value decomposition (SVD) analysis
6. 2D HD-VSFG spectrum in the ground-state bleach region at 400 fs
7. Fresnel factor correction on the $Im\chi^{(2)}$ and $\Delta Im\chi^{(2)}$ spectra of the air/water interface

Supplementary Note 1: Instrumental response of the TR-HD-VSFG setup

For evaluating the instrument response function (IRF), the intensity of third-order nonlinear optical signal corresponding to the sum of the three input beams (IR pump: ω_{pump}, IR probe: ω_{IR}, visible probe: ω_{vis}) from the air/z-cut quartz interface was measured with varying IR pump-IR probe delay as shown in Fig. S1. Because the pulse duration of the visible probe pulse (λ=795 nm) is much longer than those of IR pump and IR probe pulses, the temporal profile of the third order signal corresponds to the cross-correlation between the IR pump and IR probe pulses. The temporal profiles of the third-order signals were fit with the Gaussian function, and the obtained full-width at half-maximum (FWHM) values of IRF are listed in Table 1. The FWHM of the temporal profile ranges from 157 ± 1 to 211 ± 9 fs, depending on the IR pump frequency, and the averaged value is ~185 fs. This variance in the IRF is due to the difference in the phase-matching condition of the KTP crystal for IR pump pulse generation.

Supplementary Table 1. FWHM of the IRF obtained from fitting with Gaussian function.

Pump IR frequency (cm^{-1})	FWHM of IRF (fs)
3200	157 ± 1
3300	175 ± 3
3400	187 ± 3
3500	176 ± 2
3600	206 ± 3
3700	211 ± 9

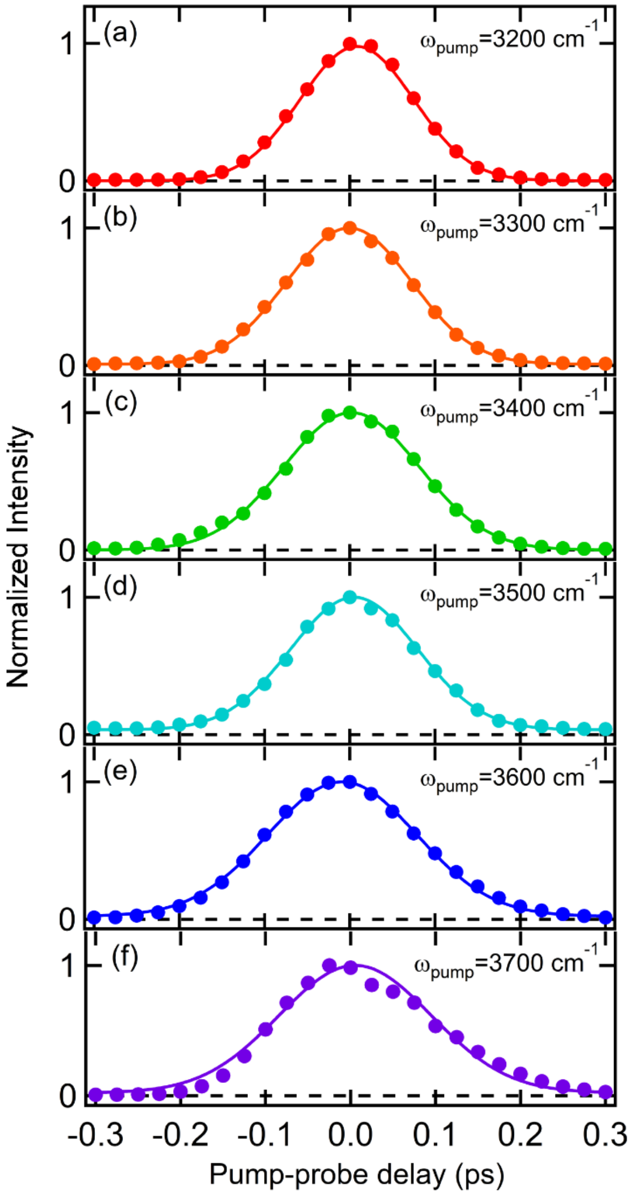

Supplementary Figure 1. Instrumental response of the TR-HD-VSFG measurements. Intensity of non-resonant third-order nonlinear optical response versus IR pump -IR probe delay measured from the air/z-cut quartz interface for pump frequencies of (a) 3200 cm^{-1}, (b) 3300 cm^{-1}, (c) 3400 cm^{-1}, (d) 3500 cm^{-1}, (e) 3600 cm^{-1}, and (f) 3700 cm^{-1}. Solid lines are the best fits with the Gaussian function.

Supplementary Note 2: Time-resolved $\Delta\text{Im}\chi^{(2)}$ spectra at long delay times after IR excitation

We measured time-resolved $\Delta\text{Im}\chi^{(2)}$ spectra of the air/water interface at long delay times to know their long-term temporal evolution. The $\Delta\text{Im}\chi^{(2)}$ spectra measured with 3300-cm^{-1} excitation are shown in Fig. S2. As seen, the spectra after 1.5 ps mainly exhibit the low-frequency part of the thermalized signal, but a small negative signal centered at around 3050 cm^{-1} is also recognized. Because this negative signal remains even after the thermalization process is completed, we concluded that this signal is not related to the vibrational dynamics of the OH stretch.

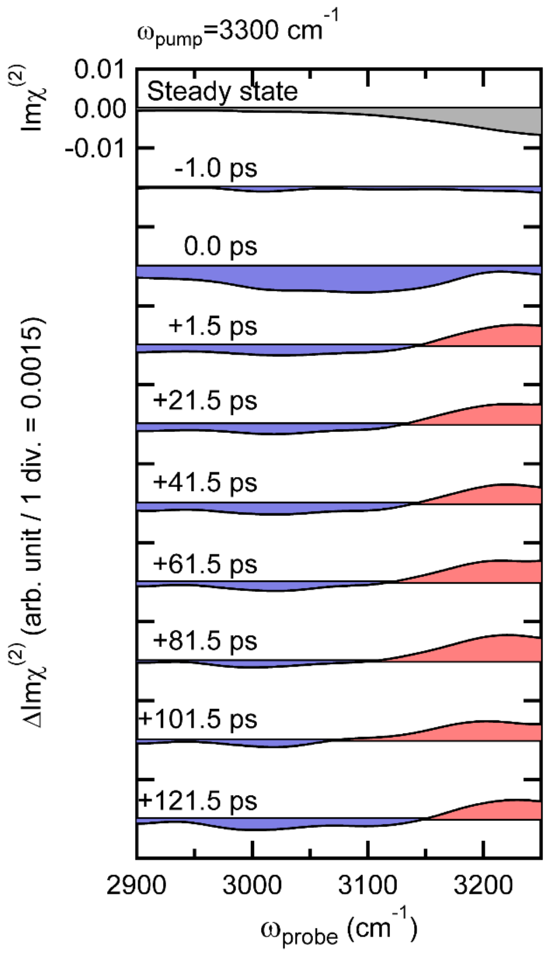

Supplementary Figure 2. Time-resolved $\Delta\text{Im}\chi^{(2)}$ spectra of the air/water interface up to the delay time of 121.5 ps measured with 3300-cm^{-1} excitation. The red and blue shaded areas indicate positive and negative signs of the $\Delta\text{Im}\chi^{(2)}$ spectra, respectively. 1 div. indicates 1 division of y-axis.

Supplementary Note 3: Details of the fitting analysis of the temporal traces of the ΔImχ$^{(2)}$ signals

3.1. Fitting for the temporal traces of ΔImχ$^{(2)}$ with direct excitation of HB OH stretch

The IR pump pulses centered at 3200, 3300, 3400, and 3500 cm^{-1} directly populate the first excited state of the HB OH stretch. Thus, the excited-state population dynamics can be simply represented with single exponential decay, i.e., Eq. (2) in the main text. In the actual fitting, we used the following fitting function:

$$F(t)=\int_{-\infty}^{\infty}\frac{1}{\sqrt{2\pi\sigma^2}}\exp\left(-\frac{(t'-t)^2}{2\sigma^2}\right)\left(f(t')+f_{\infty}(t')\right)dt'+y_0,$$

$$\text{where}\quad f(t)=\begin{cases}A\exp\left(-\dfrac{t-x_0}{T_{1,HB}}\right), & \text{if } t\geq 0\\ 0, & \text{if } t<0\end{cases}, \qquad \text{(S1)}$$

$$f_{\infty}(t)=\begin{cases}C_{\infty}\exp\left(-(t-x_0)/T_{\infty}\right), & \text{if } t\geq 0\\ 0, & \text{if } t<0\end{cases}.$$

Here, σ is a value associated with the FWHM of IRF (FWHM$=2\sqrt{2ln2}\sigma$), and A is the amplitude of the transient signal. x_0 is the temporal offset that was introduced to compensate for the shift of the time origin due to the long-term accumulation (~10 hours), which we allowed to change within ±0.025 ps in the fitting. y_0 is the amplitude offset to compensate for the background fluctuation. As described in the previous section, a long-lasting negative feature remains even after the thermalization process is finished (Fig. S2). This component is not related to the vibrational dynamics of the interfacial water. We treated it as $f_{\infty}(t)$. C_{∞} is its amplitude, and its time constant T_{∞} was set at 1000 ps. The fitting parameters, including the evaluated T_1 time of the HB OH stretch, $T_{1,\mathrm{HB}}$, are listed in Table S2.

3.2. Fitting for the temporal traces of ΔImχ$^{(2)}$ with excitations of free OH stretch

The IR pump pulse centered at 3700 cm^{-1} selectively populates the first excited states of the free OH stretch, while the IR pump centered at 3600 cm^{-1} populates the first excited states of both HB- and free OH stretches. Thus, the excited-state population dynamics of the HB OH stretch observed with 3700- and 3600-cm^{-1} excitations are represented as Eq. (6) in the main text. In the actual fitting, we used the following fitting function:

$$F(t)=\int_{-\infty}^{\infty}\frac{1}{\sqrt{2\pi\sigma^2}}\exp(-\frac{(t^{'}-t)^2}{2\sigma^2})(f(t^{'})+f_{\infty}(t^{'}))dt^{'}+y_0,$$

$$\text{where } f(t)=\begin{cases}Ar\exp(-\frac{t-x_0}{T_{1,HB}})+B(1-r)\frac{T_{1,HB}}{T_{1,HB}-T_{1,free}}[-\exp(-\frac{t-x_0}{T_{1,free}})+\exp(-\frac{t-x_0}{T_{1,HB}})],\ \text{if } t\geq 0\\ 0,\ \text{if } t<0\end{cases},\quad \text{(S2)}$$

$$f_{\infty}(t)=\begin{cases}C_{\infty}\exp(-(t-x_0)/T_{\infty}),\ \text{if } t\geq 0\\ 0,\ \text{if } t<0\end{cases},$$

where the meanings of σ, x_0, y_0 C_∞, and T_∞ are the same as those in Eq. (S1). Also in this fitting, we allowed the temporal offset x_0 to change within ±0.025 ps. A and B are the amplitudes of the transient signals originating from the directly excited HB OH stretch and those of the excited HB OH stretch converted from the excited free OH stretch, respectively. r is the fraction of the HB OH excited directly by the IR pump pulse.

By using the fitting function above, the temporal traces obtained with 3600-cm^{-1} (Fig. 4a in the main text) and 3700-cm^{-1} (Fig. 4b) excitations were globally fitted by treating r as a free parameter between 0 and 1 (for 3600-cm^{-1} excitation) and fixing at 0 (for 3700-cm^{-1} excitation), respectively, with use of common $T_{1,\mathrm{free}}$ and $T_{1,\mathrm{HB}}$ values. All parameters of the best fits are listed in Table 3, and $T_{1,\mathrm{free}}$ = 0.84 ± 0.08 ps and $T_{1,\mathrm{HB}}$ = 0.34 ± 0.06 ps were obtained.

Supplementary Table 2. Fitting result for the temporal traces of $\Delta\mathrm{Im}\chi^{(2)}$ signals in Fig. 3 of the main text.

Fitting parameters	Pump IR frequency (cm^{-1})			
	3200	3300	3400	3500
A (a. u.)	0.048 ± 0.003	0.063 ± 0.008	0.064 ± 0.005	0.078 ± 0.005
$T_{1,\mathrm{HB}}$ (ps)	**0.28 ± 0.03**	**0.24 ± 0.05**	**0.32 ± 0.06**	**0.29 ± 0.04**
σ (ps)†	0.066	0.075	0.080	0.075
y_0 (a. u.)	0.002 ± 0.002	0.004 ± 0.004	-0.001 ± 0.003	0.002 ± 0.003
x_0 (ps)	-0.025 ± 0.012	-0.025 ± 0.019	-0.025 ± 0.013	-0.025 ± 0.010
C_∞ (a. u.)	0.003 ± 0.002	0.013 ± 0.005	0.017 ± 0.004	0.014 ± 0.003

†σ is determined from the measurement of cross correlation between the pump and the probe IR beams and the parameter is fixed during the fitting procedure. FWHM of the cross-correlation is $2\sqrt{2ln2}\sigma$.

Supplementary Table 3. Fitting result for the temporal traces of $\Delta\mathrm{Im}\chi^{(2)}$ signals in Fig.4 of the main text.

Fitting parameters	Pump IR frequency (cm^{-1})	
	3600	3700
A (a. u.)	0.12	0
B (a. u.)	0.22	0.18
$T_{1,free}$ (ps)	**0.84 ± 0.08**	**0.84 ± 0.08**
$T_{1,HB}$ (ps)	**0.34 ± 0.06**	**0.34 ± 0.06**
σ (ps)	0.090	0.090
r (a. u.)	0.36 ± 0.07	0
y_0 (a. u.)	0.002 ± 0.002	-0.007 ± 0.004
x_0 (ps)	0.025 ± 0.063	0.025 ± 0.043
C_∞ (a. u.)	-0.002 ± 0.004	0.002 ± 0.005

Supplementary Note 4: Singular value decomposition (SVD) analysis of the time-resolved $\Delta \mathrm{Im}\chi^{(2)}$ spectra in the excited-state HB OH stretch band region

To check the robustness of the T_1 time values determined directly by the temporal change of the excited-state HB OH stretch signal, we also performed SVD analysis of the $\Delta \mathrm{Im}\chi^{(2)}$ spectra. Details of the SVD analysis have been described elsewhere (e.g., Ref. 44 in the main text). Briefly, the SVD analysis decomposes a set of time-resolved spectra into singular values, spectral components, and corresponding temporal traces. The matrix representation of this decomposition is given as follows,

$$M = UWV^T = \begin{pmatrix} \overrightarrow{u_1} & \overrightarrow{u_2} & \cdots \end{pmatrix} \begin{pmatrix} w_1 & 0 & \cdots \\ 0 & w_2 & \\ \vdots & & \ddots \end{pmatrix} \begin{pmatrix} \overrightarrow{v_1}^T \\ \overrightarrow{v_2}^T \\ \vdots \end{pmatrix}, \tag{S3}$$

where M is an m x n matrix of the input time-resolved spectra, of which m is the number of data points in each spectrum and n is the number of delay times. The m x n matrix of U and n x n matrix V^T correspond to the decomposed spectral components and their temporal traces, respectively, and U and V^T consist of orthogonal vectors $\vec{u}$ and $\vec{v}^T$. W is an n x n matrix of singular value w. The powerfulness of the SVD analysis is that it provides the number of independent spectral components (i.e., the number of the significant singular value) that are needed to reconstruct all the input time-resolved spectra, free from any model. Nevertheless, because the SVD analysis is a purely mathematical procedure, its direct outputs of the spectral components ($\vec{u}$) and temporal traces ($\vec{v}^T$) do not have any physical meanings as they are. Thus, it is necessary to find appropriate linear combinations of the direct outputs of the spectral components and temporal traces by introducing reasonable assumptions or models, to obtain the actual spectral components and their temporal traces.

First, we performed the SVD analysis on the $\Delta \mathrm{Im}\chi^{(2)}$ spectra in the probing region from 2900 cm^{-1} to 3050 cm^{-1}, which is the same frequency range for the signal integration to obtain the temporal traces analyzed in the main text. We applied the SVD analysis to the $\Delta \mathrm{Im}\chi^{(2)}$ spectra themselves for the data obtained with excitations at 3200, 3300, 3400, 3500, and 3600 cm^{-1}. For the data obtained with 3700 cm^{-1} excitation, we subtracted the $\Delta \mathrm{Im}\chi^{(2)}$ spectrum at -

3.0 ps ($\Delta\text{Im}\chi^{(2)}$(t) - $\Delta\text{Im}\chi^{(2)}$(-3.0 ps)) before the SVD analysis because the transient signal amplitude is low and comparable to the background level seen at -3.0 ps.

Fig. S3 (cross marks) shows the singular values obtained, and there is only one significant singular value for the $\Delta\text{Im}\chi^{(2)}$ spectra measured with each excitation. It indicates that this probing region only contains one single spectral component, verifying the assumption of our analysis in the main text, i.e., only the excited-state HB OH band appears in the $\Delta\text{Im}\chi^{(2)}$ spectra below 3050 cm^{-1}. Fig. S4 shows the direct SVD output of traces ($\vec{v}_1^T$). Because there is only one significant singular value (w_1), the $\vec{v}_1^T$ itself represents a physically meaningful temporal trace, i.e., the population dynamics of the excited state of the HB OH stretch. Indeed, the temporal traces of $\vec{v}_1^T$ shown in Fig. S4 are almost the same as those obtained from the integrated $\Delta\text{Im}\chi^{(2)}$ signal shown in Figs. 3 and 4 of the main text.

Next, we performed SVD analysis on the $\Delta\text{Im}\chi^{(2)}$ spectra in the whole probe frequency region from 2900 cm^{-1} to 3250 cm^{-1}. Fig. S3 (open circles) shows the singular value obtained, and it indicates two significant singular values for the $\Delta\text{Im}\chi^{(2)}$ spectra measured with each excitation. It implies that the $\Delta\text{Im}\chi^{(2)}$ spectra in this probe region are reproduced with two spectral components. Then, Eq. S3 is simplified in the following form:

$$M = \begin{pmatrix} \vec{u}_1 & \vec{u}_2 \end{pmatrix} \begin{pmatrix} w_1 & 0 \\ 0 & w_2 \end{pmatrix} \begin{pmatrix} \vec{v}_1^{\,T} \\ \vec{v}_2^{\,T} \end{pmatrix} \tag{S4}$$

As mentioned at the beginning, the direct output of the SVD analysis, i.e., spectral components $\vec{u}_i$ and temporal traces $\vec{v}_i^{\,T}$, are orthogonalized as vectors, so we need to obtain adequate linear combinations to represent physically meaningful spectral components. It seemed natural to consider that the two physically meaningful spectral components are the excited-state band of the HB OH stretch and the thermalized signal. (It turned out later that this idea is too simple and incorrect. Vide infra.) Therefore, we assumed that the dynamics of the excited-state HB-OH band (*f*(t) in Eqs. S1 and S2) and that of the thermalized signal, are represented with the linear combinations of the direct output temporal traces $\vec{v}_1{}^T$ and $\vec{v}_2{}^T$, as follows,

$$\begin{pmatrix} \vec{v}_1^{\,T} \\ \vec{v}_2^{\,T} \end{pmatrix} = C \begin{pmatrix} f(t) \\ 1-\exp(-t/T_{th}) \end{pmatrix} * \exp(-t^2/2\sigma^2),$$
$$C = \begin{pmatrix} c_{11} & c_{12} \\ c_{21} & c_{22} \end{pmatrix}, \qquad \text{(S5)}$$

Note that Eq. S5 includes a factor representing the instrumental response ($\exp(-t^2/2\sigma^2)$). C is a square transformation matrix consisting of four parameters (c_{11}, c_{12}, c_{21}, and c_{22}), T_{th} is the rise time of the thermalization band, and the asterisk symbol is a convolution operator. We searched the transformation matrix C that best satisfies Eq. S5 by fitting. Then, using the best-fit C, we obtained the temporal trace of the excited-state band of the HB OH stretch ($\vec{v_1'}^{T}$) and that of thermalized signal ($\vec{v_2'}^{T}$):

$$\begin{pmatrix} \vec{v'}_1^{\,T} \\ \vec{v'}_2^{\,T} \end{pmatrix} = C^{-1} \begin{pmatrix} \vec{v}_1^{\,T} \\ \vec{v}_2^{\,T} \end{pmatrix} \qquad \text{(S6)}$$

Fig. S5 shows the temporal traces of $\vec{v_1'}^{T}$ (red circle) and $\vec{v_2'}^{T}$ (blue circle).

In the process of obtaining the best-fit C, we obtained $T_{1,\text{HB}}$, $T_{1,\text{free}}$, and T_{th}. The obtained $T_{1,\text{HB}}$ upon direct HB OH excitations are 0.20 ± 0.02, 0.29 ± 0.03, 0.34 ± 0.03, and 0.33 ± 0.03 ps for 3200-, 3300-, 3400-, and 3500-cm^{-1} excitations, respectively. As for the 3600- and 3700-cm^{-1} excitations, the $T_{1,\text{HB}}$ and $T_{1,\text{free}}$ obtained are 0.37 ± 0.06 ps and 0.82 ± 0.20 ps for the 3600-cm^{-1} excitation, and 0.33 ± 0.20 ps and 0.97 ± 1.17 ps for the 3700-cm^{-1} excitation, respectively. The blue and red solid lines plotted in Fig. S5 are the curves calculated using these values. Figs. S6 show spectral decompositions into the excited-state HB stretch band (red line) and thermalized signal (blue line), which were done based on the SVD analysis. The sums of the two spectral components (black line) very well reproduce the experimental spectra (filled area).

Although the sum of the two spectral components and their temporal traces obtained from the SVD analysis look to almost perfectly reproduce the experimental spectra, the spectra in Fig. S6 show that there is a problem and limitation for the SVD analysis. For example, the $\Delta\text{Im}\chi^{(2)}$ spectra measured with 3200-cm^{-1} (Fig. S6(a)) and 3300-cm^{-1} (Fig. S6(b)) excitations clearly exhibit a positive signal in the high-frequency region at around 3200 cm^{-1} immediately after excitation. This positive signal is attributable to the bleach of the ground-state HB OH

stretch band, which quickly disappears from the probe frequency region due to spectral diffusion. In addition, all the $\Delta\mathrm{Im}\chi^{(2)}$ spectra exhibit weak, long-lived compoments in low-frequency region, which cannot be attributable to the thermalized signal (See also Section S5 in Supplementary Information.) In this sense, the $\Delta\mathrm{Im}\chi^{(2)}$ spectra contain not only two components as the SVD analysis indicates but more spectral components. However, the spectra of the bleach of the ground-state HB OH stretch and the thermalized signal are similar to each other in the probed frequency region. (Note the very large amplitude of the temporal trace of the thermalized signal obtained with SVD at around the time origin for the 3200-cm^{-1} excitation (Fig. S5(a))). Also, the temporal behaviors between the thermal signal and long-lived component are difficult to be distinguished in the measured detay time region. As a result, anyhow, the sum of only two spectral components can reproduce the experimental data but it is not relevant in a strict sense. Therefore, although the $T_{1,HB}$ obtained by the SVD analysis are comparable to those directly determined from the experimentally observed excited-state HB OH signal described in the main text, we can safely consider that the latter values are more reliable. In fact, the $T_{1,\,HB}$ value for 3200-cm^{-1} excitation obtained with the SVD analysis (0.20 ± 0.02 ps) noticeably deviates from the $T_{1,HB}$ value obtained by the analysis described in the main text (0.28 ± 0.03 ps). This problem of the SVD analysis also indicates difficulty in the analysis of the ground-state bleach region, where the bleach of the HB OH stretch and its spectral diffusion, the excited-state HB OH stretch band, and the thermalized band appear.

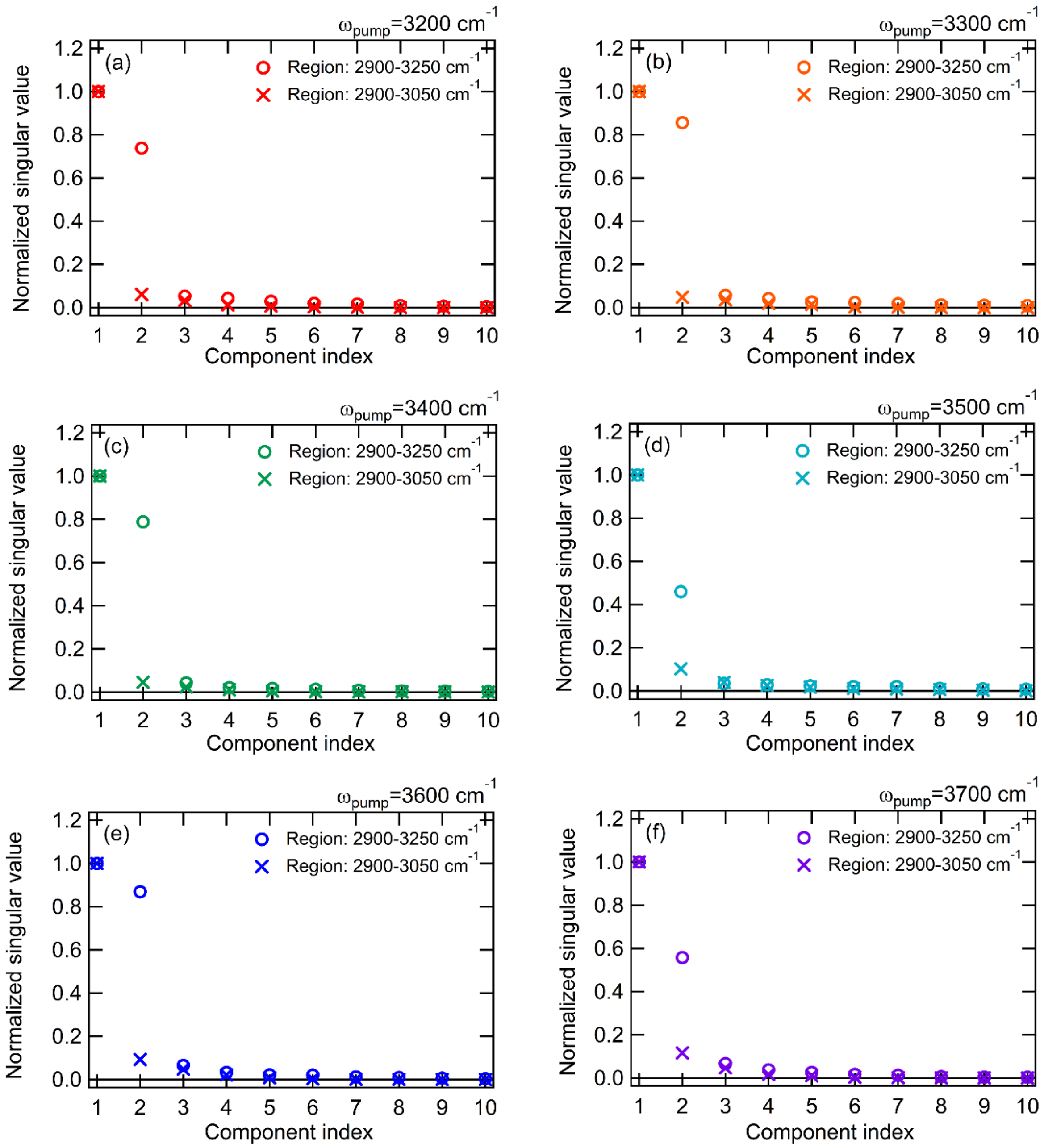

Supplementary Figure 3. Singular values obtained from SVD analysis. Singular values obtained from SVD analysis on the $\Delta \mathrm{Im}\chi^{(2)}$ spectra measured with excitations at (a) 3200 cm^{-1} (red), (b) 3300 cm^{-1} (orange), (c) 3400 cm^{-1} (green), (d) 3500 cm^{-1} (cyan), (e) 3600 cm^{-1} (blue), and (f) 3700 cm^{-1} (purple). The singular values obtained from the analysis on the whole range (2900-3250 cm^{-1}, open circle) of the $\Delta \mathrm{Im}\chi^{(2)}$ spectra and those from the low-frequency side (2900-3050 cm^{-1}, cross mark) are plotted.

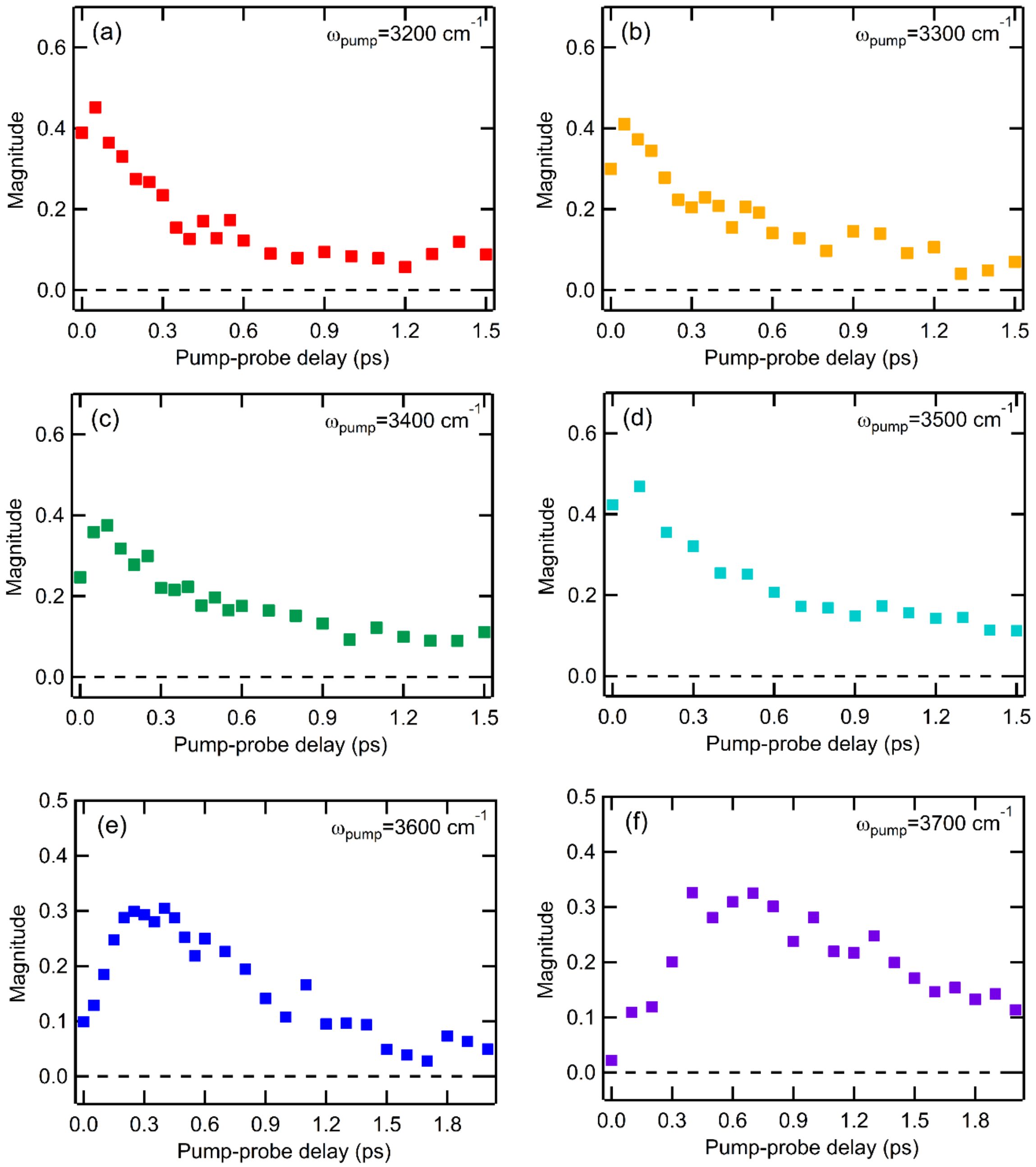

Supplementary Figure 4. Temporal traces of excited-state band of HB OH stretch obtained as the direct output of SVD analysis. The first component of the direct output temporal trance $\vec{v}_1^{\,T}$ obtained from the SVD analysis of $\Delta \mathrm{Im}\chi^{(2)}$ spectra in the IR probe range of 2900-3050 cm^{-1} measured with excitations at (a) 3200-cm^{-1}, (b) 3300-cm^{-1}, (c) 3400-cm^{-1}, (d) 3500-cm^{-1}, (e) 3600-cm^{-1}, and (f) 3700-cm^{-1}. Note that the SVD analysis only provide a single significant singular value, and hence that the direct outputs provide physically meaningful temporal traces.

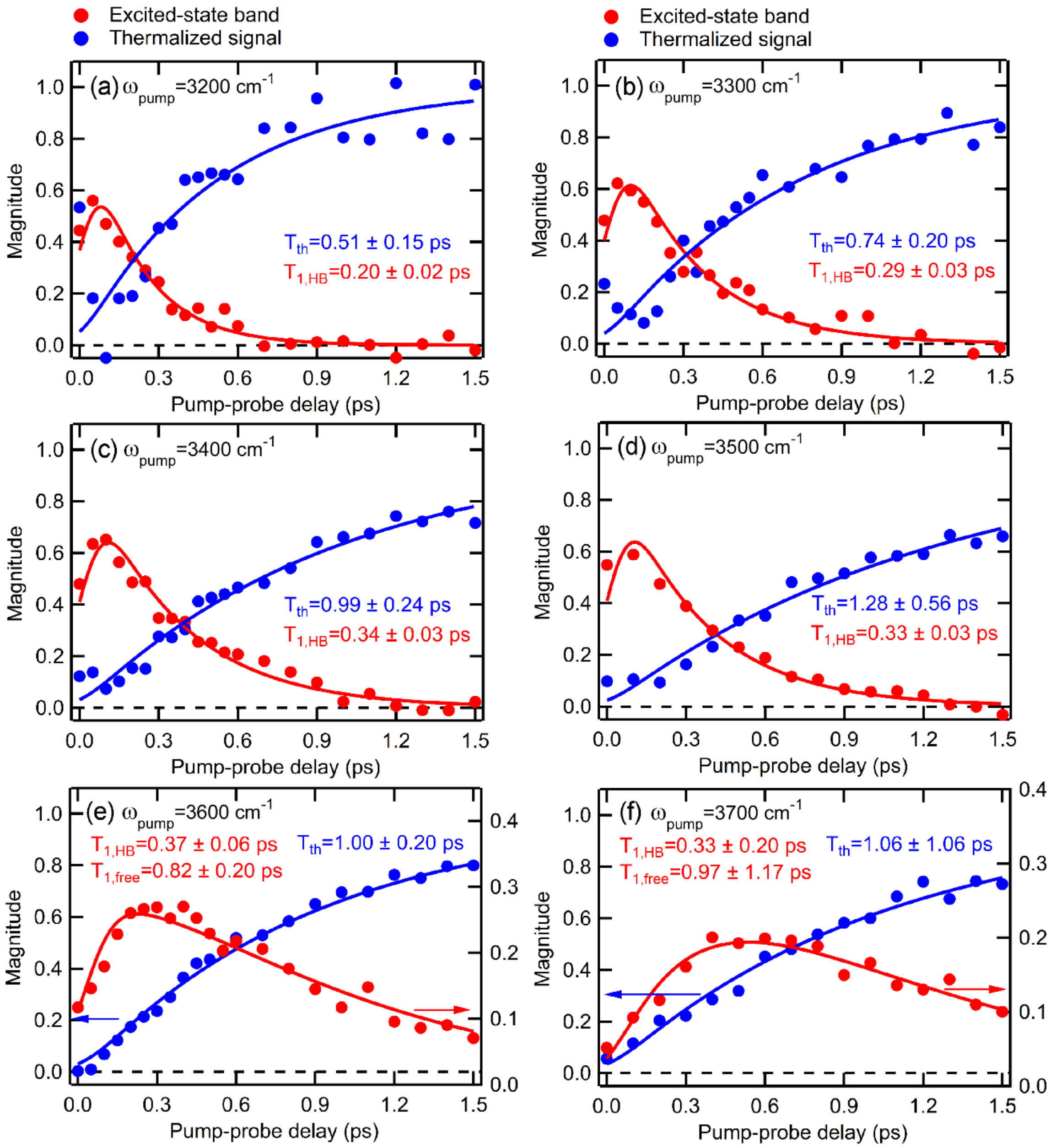

Supplementary Figure 5. Temporal traces of the excited-state HB OH stretch and thermalized signals obtained with SVD analysis. Temporal traces of the excited-state band of HB OH stretch $\overrightarrow{\boldsymbol{v}_1'}^{\boldsymbol{T}}$ (red circle) and thermalized signal $\overrightarrow{\boldsymbol{v}_2'}^{\boldsymbol{T}}$ (blue circle) obtained from the SVD analysis on the $\Delta\mathrm{Im}\chi^{(2)}$ spectra in the IR probe range of 2900-3250 cm^{-1} measured with excitations at (a) 3200 cm^{-1}, (b) 3300 cm^{-1}, (c) 3400 cm^{-1}, (d) 3500 cm^{-1}, (e) 3600 cm^{-1}, and (f) 3700 cm^{-1}. All the temporal traces are transformed from the direct output of the SVD analysis (See the text). Solid lines: curves calculated using the parameters obtained in the fitting.

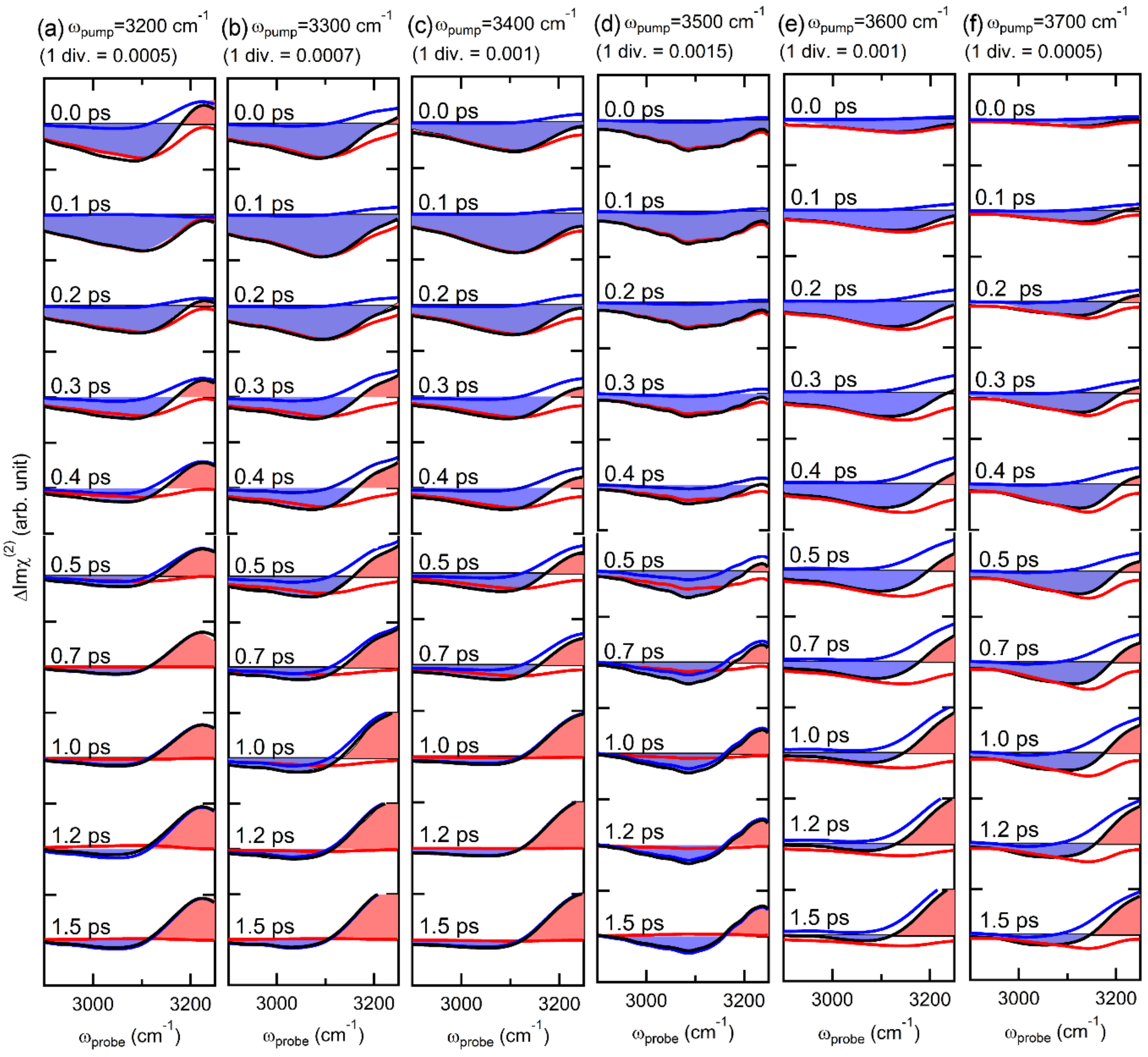

Supplementary Figure 6. Spectral decomposition of $\Delta\text{Im}\chi^{(2)}$ spectra based on the SVD analysis in the IR probe range of 2900-3250 cm^{-1}. Excited-state HB OH band (red), thermalized signal (blue), their sum (black), and experimental data (shaded area). The IR pump frequencies are (a) 3200 cm^{-1}, (b) 3300 cm^{-1}, (c) 3400 cm^{-1}, (d) 3500 cm^{-1} (e) 3600 cm^{-1}, and (f) 3700 cm^{-1}.

Supplementary Note 5: TR-HD-VSFG experiments using broadband excitation in the ground-state bleach region and singular value decomposition (SVD) analysis

To obtain more information about the thermalized signal, we performed TR-HD-VSFG measurements in the ground-state bleach region. In this experiment, we used a broadband IR pump pulse that can excite the whole broad HB OH stretch band to minimize the spectral evolution due to spectral diffusion. The broadband IR pump pulse was generated through self-phase modulation by loosely focusing the narrow-band IR pump pulse (ω_{pump} ~3400 cm^{-1}) onto 2 mm YAG (111) and 1 mm silicon plates, and it was spatially filtered by an iris after the collimating lens.

Figure S7(a) shows the obtained $\Delta\mathrm{Im}\chi^{(2)}$ spectra, which show both ground-state bleach (3200-3550 cm^{-1}) and excited-state HB OH (2900-3200 cm^{-1}) bands immediately after IR excitation. The $\Delta\mathrm{Im}\chi^{(2)}$ spectrum exhibits a noticeable spectral change in the sub-picosecond time region. After 1 ps, the $\Delta\mathrm{Im}\chi^{(2)}$ spectra consist mostly of the thermalized signal exhibiting positive and negative spectral features below and above ~3500 cm^{-1}, respectively, which is consistent with our previous TR-HD-VSFG study carried out with narrowband excitation (Ref. 43 in the main text). As seen, the spectrum of the thermalized signal does not exhibit any signal in the frequency region below 3100 cm^{-1}.

When we perform TR-HD-VSFG measurements in this ground-state bleach region using a narrow-band IR pump pulse, we observe ultrafast spectral diffusion (Ref. 43 in the main text). Thus, we cannot apply SVD analysis to the time-resolved spectra because SVD cannot reasonably treat gradual spectral change such as spectral diffusion. However, the broadband excitation employed in this experiment does not create a spectral hole in the broad HB OH stretch band and hence suppresses spectral diffusion. Furthermore, the present study on the excited-state HB OH signal revealed that the T_1 time of HB OH stretch is rather insensitive to the pump frequency. Thus, the time-resolved $\mathrm{Im}\chi^{(2)}$ spectra obtained with broadband excitation are expected to simply be represented by the linear combinations of the two spectral components, i.e., (1) ground-state bleach & excited-state HB OH stretch bands and (2) thermalized signal. Therefore, we performed SVD analysis and actually obtained only two major singular values that correspond to these two spectral components (Fig. S7(b)). In the same way as the SVD analysis described in section S4, we applied model fitting using

exponential decay and rise to obtain a transformation matrix *C*, and obtained spectral components and temporal traces of the excited-state population of the HB OH stretch and thermalized signal by using this *C* (Fig. S7(c) and (d)). The spectral component consisting of the ground-state bleach & excited-state HB OH stretch bands decay with a time constant of 0.34 ± 0.05 ps, which accords very well with the T_1 time of the HB OH stretch determined from the temporal traces of the excited-state signal in the low-frequency region in the present study (Fig. 5 of the main text). On the other hand, the rise time of the spectral component corresponding to the thermalized signal is determined to be 0.85 ± 0.28 ps, and this time constant at the air/water interface is very similar to the rise time of the thermalized signal in bulk water (700-800 fs) which was determined by time-resolved IR experiments (Ref. 5 and 6 in the main text).

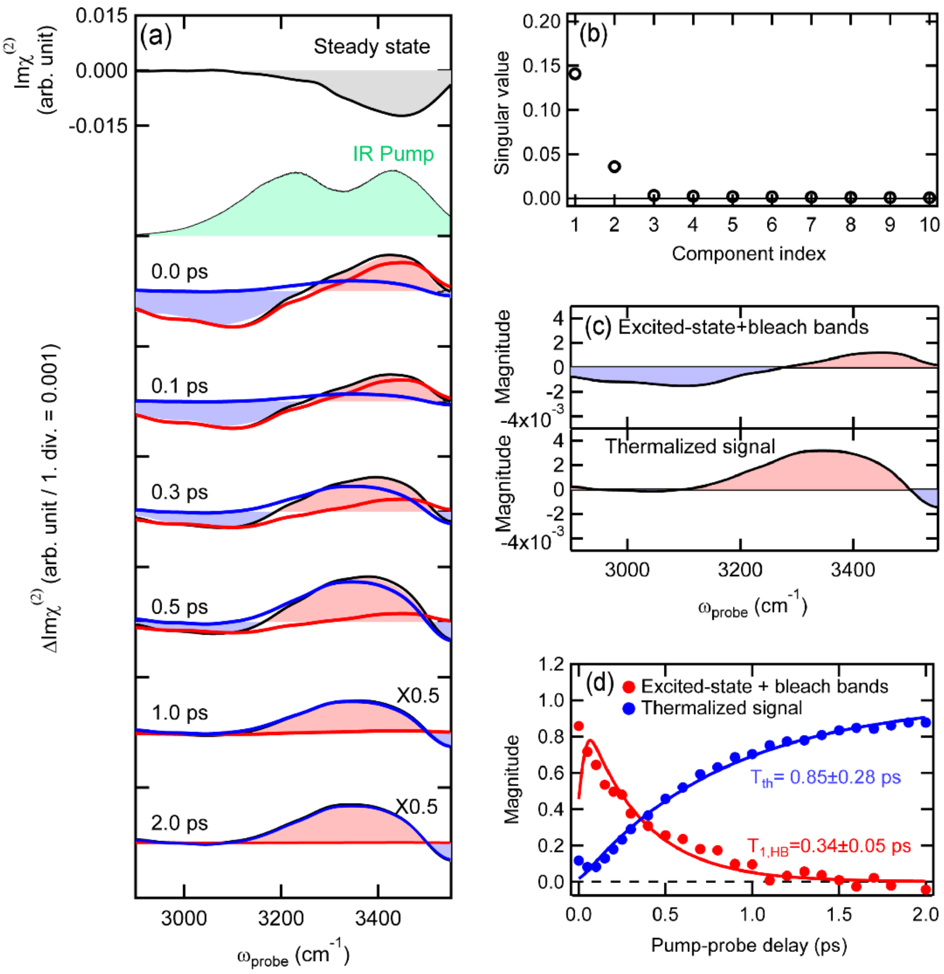

Supplementary Figure 7. Time-resolved ΔImχ$^{(2)}$ spectra in the 2900 - 3550 cm^{-1} region and the SVD analysis. (a) ΔImχ$^{(2)}$ spectra in the IR probe range of 2900 - 3550 cm^{-1} and the spectral decomposition based on SVD. Experimental data (shaded area), decomposed spectral components due to the excited-state transition + groud-state bleach bands of the HB OH stretch (red line), thermalized signal (blue line), and their sum (black line). The spectral profile of the broadband IR pump is shown on top of the ΔImχ$^{(2)}$ spectra. (b) Singular values obtained from the SVD analysis. (c) Spectral components due to excited-state and ground-state bleach bands (top) and thermalized signal (bottom). (d) Temporal traces of the two decomposed spectral components.

Supplementary Note 6: 2D HD-VSFG spectrum in the ground-state bleach region at 400 fs

To confirm the ultrafast spectral diffusion of the HB OH stretch at the air/water interface, we performed a 2D HD-VSFG measurement for the ground-state bleach region of the HB OH stretch (3200 - 3550 cm^{-1}) at 400 fs. The details of our 2D HD-VSFG setup and measurements have been reported elsewhere (Ref. 40 in the main text). Briefly, we measured $\Delta Im\chi^{(2)}$ spectra using five IR pump frequencies (ω_{pump}) at 3200 cm^{-1}, 3300 cm^{-1}, 3400 cm^{-1}, 3500 cm^{-1}, and 3600 cm^{-1}, and a 2D HD-VSFG spectrum (2D $\Delta Im\chi^{(2)}$ spectrum) was constructed by combining five $\Delta Im\chi^{(2)}$ spectra with interpolation along the ω_{pump} axis (y-axis). It is noted that the bandwidth of the IR pump used in the present measurement is narrower (~120-150 cm^{-1}) than that of the IR pump used in our first 2D HD-VSFG measurements at the air/water interface (~200 cm^{-1}) (Ref. 34 in the main text). Fig. S8(a) shows the 2D HD-VSFG spectrum obtained. The positive lobe in the 2D spectrum is nearly vertical along the ω_{pump} axis, indicating that spectral diffusion is almost completed at 400 fs. Fig. S8(b) depicts the 1D horizontal slices of the 2D HD VSFG spectrum, which correspond to $\Delta Im\chi^{(2)}$ spectra observed with the IR pump at 3300 cm^{-1} (bottom), 3400 cm^{-1} (middle), and 3500 cm^{-1} (top). These three spectra show that the bandwidth of the positive bleaching band is similar (~200 cm^{-1} FWHM) although a subtle difference is noticed for the spectrum at ω_{pump}=3500 cm^{-1}. Based on this 2D HD-VSFG spectrum at 400 fs, we can safely conclude that the time scale of the spectral diffusion at the air/water interface is shorter than or, at least, comparable to the T_1 time of the HB OH stretch determined in the present study (the main text, Tables S2, and S3). This result strongly supports our argument that ultrafast spectral diffusion largely washes out the pump frequency dependence of the T_1 time of the HB OH stretch at the air/water interface.

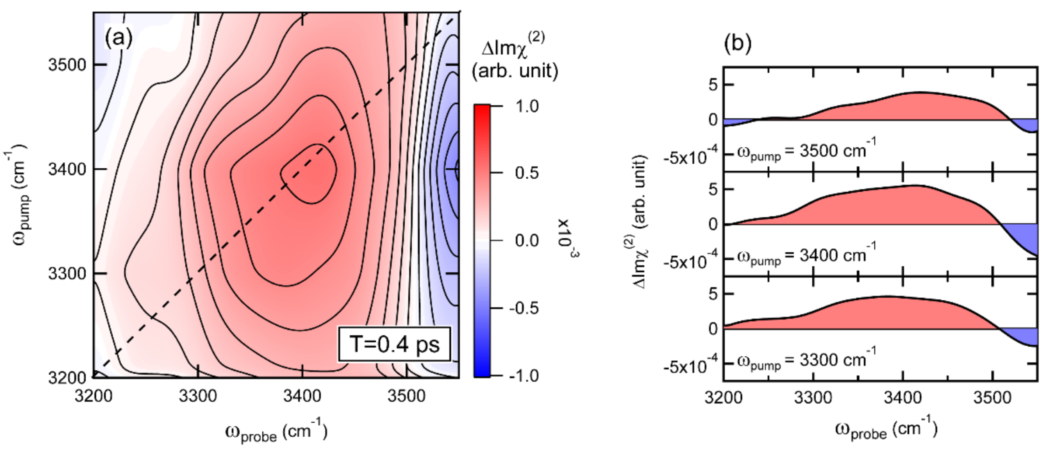

Supplementary Figure 8. 2D HD-VSFG spectrum at the air/water interface. (a) 2D HD-VSFG spectrum at the air/water interface measured at 400 fs. (b) 1D slices of the 2D HD-VSFG spectrumt at ω_p =3500 cm^{-1} (top), 3400 cm^{-1} (middle), and 3300 cm^{-1} (bottom).

Supplementary Note 7: Fresnel factor correction on the $\mathrm{Im}\chi^{(2)}$ and $\Delta\mathrm{Im}\chi^{(2)}$ spectra of the air/water interface

Fig. S9 compares $\mathrm{Im}\chi^{(2)}$ spectra at the air/water interface with and without the correction for the Fresnel factor. For the correction, we adopted the three-layer model[1] using two different refractive indices for the interface region: one is the refractive indices of bulk water ($n'=n_{H2O}$) and the other is a value in between the refractive indices of air and bulk water ($n_{air}<n'<n_{H2O}$) which is estimated by applying the Lorentz model to the interface region.[2] Since n_{H2O} in this frequency range significantly changes due to the vibrational resonance of water OH stretch, the modulation of the Fresnel factor of $L_{zz}(\omega_2)$ becomes larger as the used n' value approaches n_{H2O}. In fact, the largest effect of the Fresnel factor correction is seen when n' is set to n_{H2O} (Fig. S9(a)). Nevertheless, the effect of the correction on the $\mathrm{Im}\chi^{(2)}$ spectrum is seen only above 3100 cm^{-1} where the IR absorption due to the OH stretch is significant, and the spectral region between 2900 and 3050 cm^{-1}, where the excited-state signal of the HB OH stretch appears, is not influenced even in this case. For a more realistic refractive index of the interface ($n_{air}<n'<n_{H2O}$), the $\mathrm{Im}\chi^{(2)}$ spectrum only exhibits a subtle shift of the HB OH stretch band after Fresnel factor correction, and the change below 3100 cm^{-1} is negligible (Fig S9(b)).

Fig. S10 shows time-resolved $\Delta\mathrm{Im}\chi^{(2)}$ spectra measured with 3400-cm^{-1} excitation with and without the Fresnel factor correction. (For the convenience of comparison, we multiplied the magnitude of the $L_{yy}(\omega_{SF})L_{yy}(\omega_1)L_{zz}(\omega_2)$ making the amplitude of the $\Delta\mathrm{Im}\chi^{(2)}$ preserved.) We made the correction using the three-layer model with two interface refractive indices, as in the case of the steady-state $\mathrm{Im}\chi^{(2)}$ spectrum. Compared to the $\Delta\mathrm{Im}\chi^{(2)}$ spectra without the correction (Fig. S10 (a)), the $\Delta\mathrm{Im}\chi^{(2)}$ spectra after the correction using the two n' values only exhibit subtle changes in the frequency region above 3100 cm^{-1} (Fig.S10 (b) and (c)). Furthermore, the temporal profiles of the $\Delta\mathrm{Im}\chi^{(2)}$ signal integrated from 2900 cm^{-1} to 3050 cm^{-1} show almost no change with the Fresnel factor correction, and the evaluated $T_{1,HB}$ values are essentially the same (Fig. S11). These results of the analysis show that the effect of the Fresnel factor correction is negligible for the $\Delta\mathrm{Im}\chi^{(2)}$ spectra, temporal traces of the excited-state transition signal of the HB OH stretch, and evaluated T_1 values discussed in the main text.

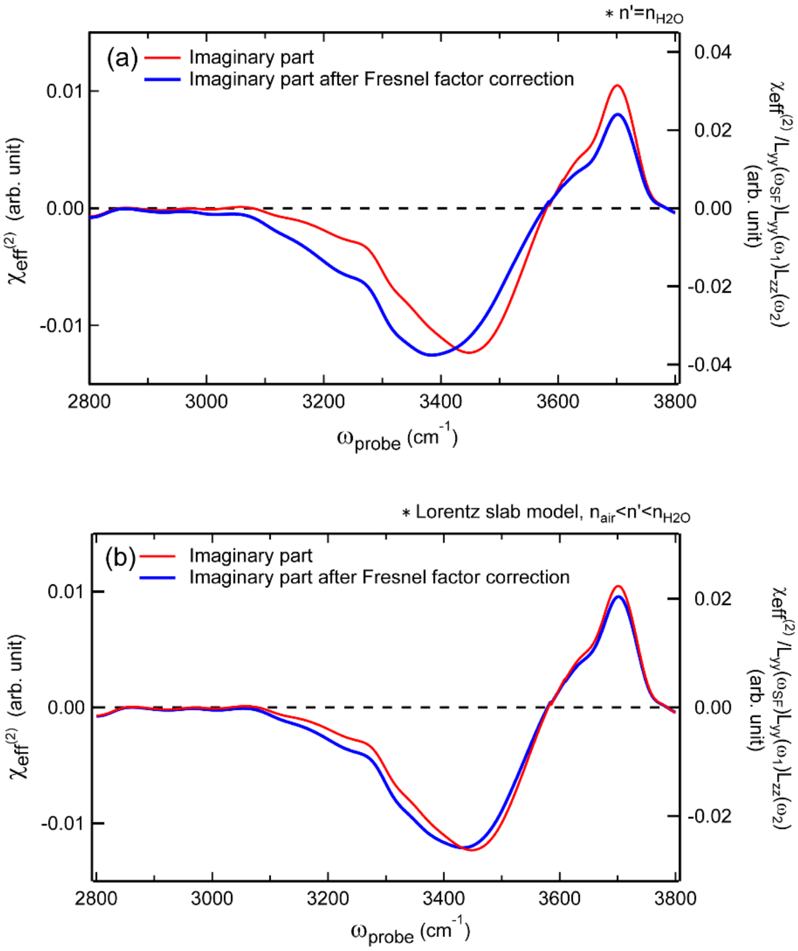

Supplementary Figure 9. Steady-state Im$\chi^{(2)}$ spectrum at the air/water interface with and without the Fresnel factor correction. (a) Im$\chi^{(2)}$ spectrum corrected with the interfacial refractive index set to the bulk water value (n'=n_{H2O}). (b) Im$\chi^{(2)}$ spectrum corrected with the interfacial refractive index estimated by applying the Lorentz model to the interface (n_{air} <n'<n_{H2O}). In both panels, Im$\chi^{(2)}$ spectrum without the correction is also shown for the comparison (red line).

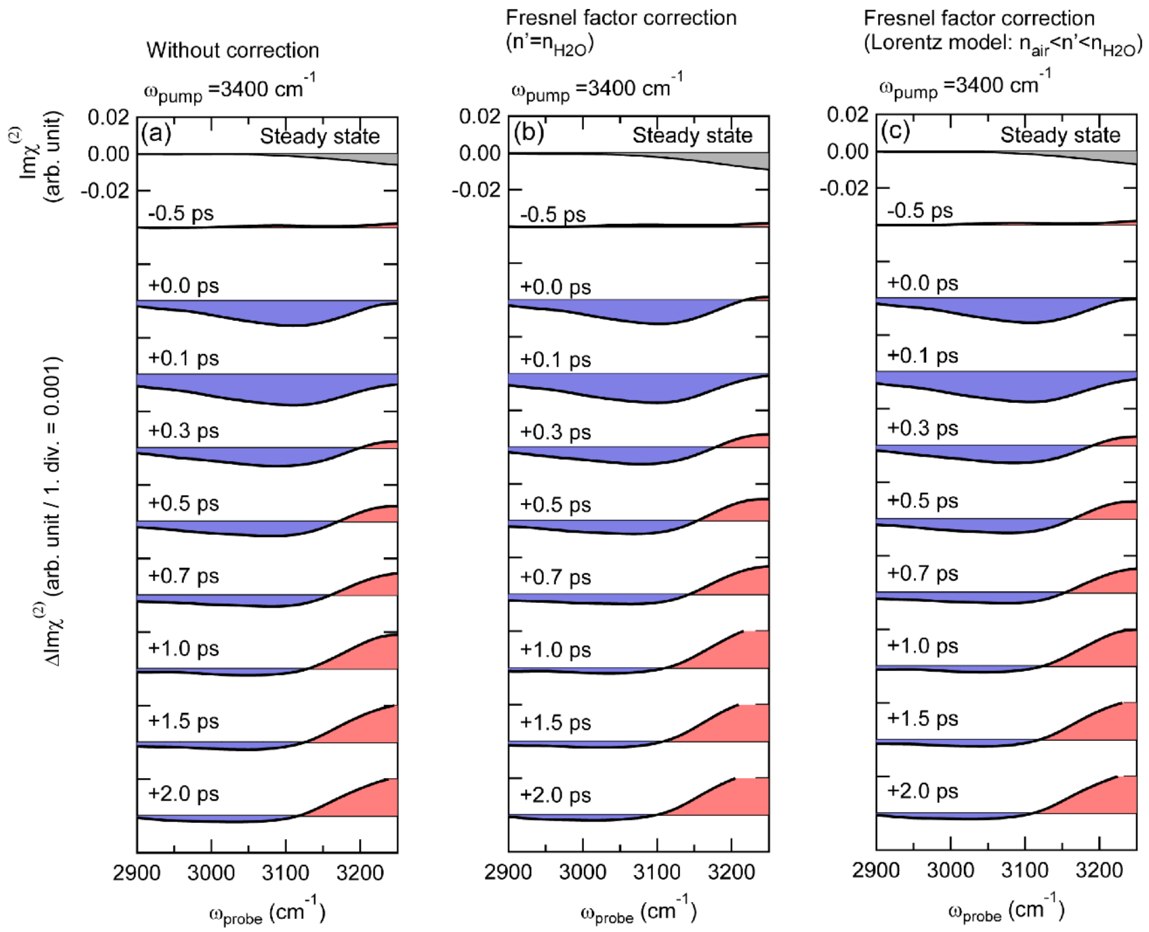

Supplementary Figure 10. Time-resolved $\Delta Im\chi^{(2)}$ spectra at the air/water interface with and without the Fresnel factor correction. (a) $\Delta Im\chi^{(2)}$ spectra without the Fresnel factor correction (same as Fig.2(c) in the main text). (b) $\Delta Im\chi^{(2)}$ spectra corrected with the interfacial refractive index set to the bulk water value (n'=n_{H2O}). (c) $\Delta Im\chi^{(2)}$ spectra corrected with the interfacial refractive index estimated by applying the Lorentz model to the interface (n_{air} <n'<n_{H2O}).

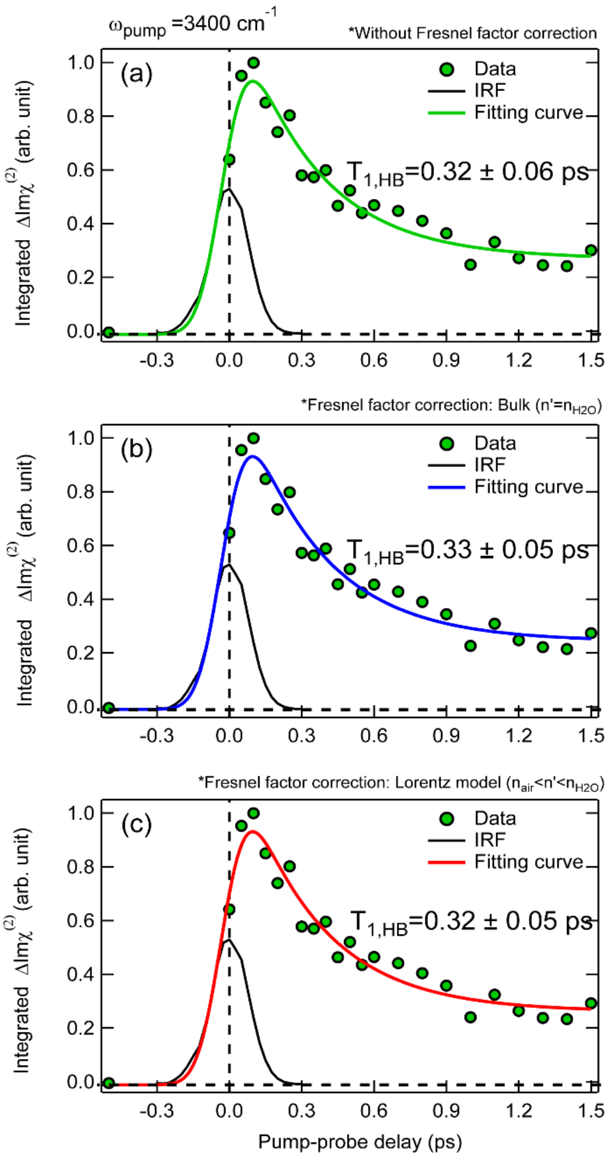

Supplementary Figure 11. Temporal traces of the ΔImχ$^{(2)}$ signal with and without the Fresnel factor correction. The ΔImχ$^{(2)}$ signal is obtained with the pump frequency at 3400 cm^{-1}. (a) Trace without Fresnel factor correction (same as Fig.3(c) in the main text). (b) Trace after the Fresnel factor correction with the interfacial refractive index set to the bulk water value (n'=n_{H2O}). (c) Trace after the Fresnel factor correction corrected with the interfacial refractive index estimated by applying the Lorentz model to the interface (n_{air} <n'<n_{H2O}).

Supplementary References

1. Shen, Y. R., *Fundamentals of Sum-frequency Spectroscopy*, Cambridge University Press (2016).

2. Zhuang X. et al. Mapping molecular orientation and conformation at interfaces by surface nonlinear optics *Phys. Rev. B* **59**, 12632 (1999).
